# A co-infection case report of *Taenia saginata* in a patient with subclinical clonorchiasis confirmed by the combination of diagnostic tools

**DOI:** 10.1186/s12879-019-3758-0

**Published:** 2019-02-06

**Authors:** Ju Hyeon Shin, Eun Jeong Won, Jee Seung Jung, Kyung-Hwa Park, Kwang Il Nam, Soo Hyun Kim, Jong Hee Shin

**Affiliations:** 1 Department of Laboratory Medicine, Chonnam National University Hospital, Gwangju, Republic of Korea; 2 Department of Parasitology and Tropical Medicine, Chonnam National University Medical School, 264, Seoyang-ro, Hwasun-eup, Hwasun-gun, Jeollanam-do, 58128 Republic of Korea; 3 Department of Infectious Diseases, Chonnam National University Hospital, Gwangju, Republic of Korea; 4 Department of Anatomy, Chonnam National University Medical School, Hwasun-gun, Jeollanam-do, Republic of Korea

**Keywords:** *Taenia saginata*, *Clonorchis sinensis*, Molecular diagnosis, Ova, ELISA

## Abstract

**Background:**

Clonorchiasis is the common parasitic infection in the general population of the Republic of Korea, however, taeniasis is scarcely reported recently. Here, we describe a case of co-infection with the cestode *T. saginata* in a patient with subclinical clonorchiasis diagnosed by a combination of diagnostic tools in Korea.

**Case presentation:**

A 56-year-old man visited the hospital having passed proglottids in his stool for the past two months and brought a stool sample with segments to our hospital. He had no abdominal symptoms, such as nausea, vomiting, abdominal pain, diarrhea, or constipation. He used to consume raw beef and fish frequently. We could not find evidence of gravid proglottids which contain fully developed uteri filled with ova or branched uterine structures, within the submitted sample. To identify the tapeworm species, we carried out molecular analyses on the proglottids. The *cox1* and *ef1a* sequences had a 100% match with those of *T. saginata* and differed from the sequences of the other *Taenia* species. Upon examination of stool samples fixed by formalin-ether concentration method, no *Taenia* species ova were observed in 10 slides. Instead, *C. sinensis* ova were observed, despite the level of IgG specific to *C. sinensis* being within the normal range. The patient was treated with praziquantel (25 mg/kg, three times a day) for 3 days, and subsequently *C. sinensis* ova were not found in his stool.

**Conclusion:**

Our case indicates that a combination of morphological, serological, and molecular diagnostic tools should be used for the accurate diagnosis of subclinical parasitic infections.

**Electronic supplementary material:**

The online version of this article (10.1186/s12879-019-3758-0) contains supplementary material, which is available to authorized users.

## Background

Intestinal parasitic infections are still a major public health issue in worldwide [1]. Until the 1970s, many Koreans had intestinal parasitic infections, mostly from soil-transmitted helminthes [2]. Although overall helminth egg-positive rate was 84.3% in the 1st Nationwide Survey [2], a dramatic decline of the egg-positive rate has been shown to 2.6% in the 8th Nationwide Survey, 2013 [3]. Thus, at present, intestinal parasitic infections are not recognized as a critical problem in Korea. The public health focus has also shifted from soil-transmitted helminthiases to food-borne parasitic infections. Of food-borne parasites, *Clonorchis sinensis* showed the highest prevalence in Korea showing 2.4% of egg-positive rate in general population [3]. The parasite is more prevalent in populations living by rivers, with an egg-positive rate of 11.4% [4], as humans usually become infected after ingesting raw or undercooked freshwater fish harboring *C. sinensis* metacercariae [5]. Human taeniasis, another food-borne parasitic infection, is a zoonotic disease, because it involves pigs (*Taenia solium* and *Taenia asiatica*) or cattle (*Taenia saginata*) as an intermediate host and humans as the definitive host [6, 7]. Studies dating back to 1924 reported *Taenia* prevalence in Korea at 7.2% or higher [8–10], but the national prevalence was 1.98% in 1971 [2]. The national prevalence continued to decline, and was reported as 0.004% in 2013 [3]. Here, we describe a case of co-infection case with the cestode *T. saginata* and the trematode *C. sinensis* by using a combination of diagnostic tools in a patient with subclinical parasitic infection.

## Case presentation

**Fig. 1:**
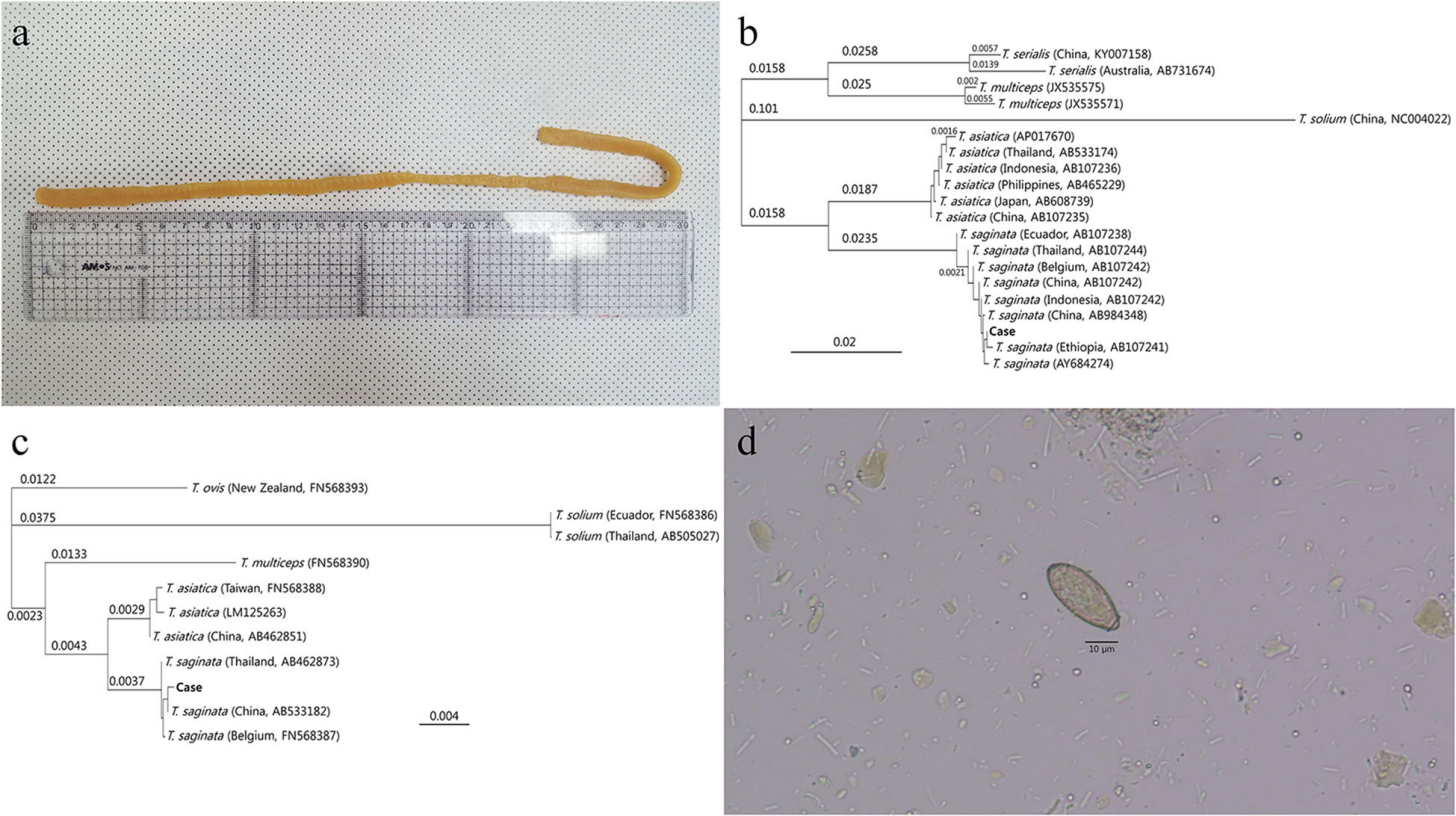
**a** Overall appearance of proglottids in the patient’s feces, about 37 cm in length. **b** Phylogenetic tree for the *cox1* gene and (**c**) *ef1a* gene obtained from the patient’s *Taenia* tapeworm, compared to other *Taenia* species in GenBank. The GenBank accession number is indicated. The scale bar indicates a sequence distance of 0.02 and 0.004 nucleotide substitutions per site. **d** Ovum of *C. sinensis* found in the patient’s stool specimen. A scale bar is included

A 56-year-old man complained of passing proglottids in his stool intermittently over the last two months. No abdominal symptoms, such as nausea, vomiting, abdominal pain, diarrhea, or constipation were present. He reported frequent consumption of raw beef and fish (both marine and freshwater fish), and had no history of traveling abroad. He had previously obtained 400 mg of albendazole from the pharmacy and taken it once orally without clinical improvement. After that, he was prescribed 600 mg of praziquantel at a local clinic and had taken it once orally as well. He brought his stool sample, which included the passed segments to our hospital (Fig. 1a). The segments were pressed between two microscope slides and examined macroscopically without staining. We could not observe gravid proglottids, which contain fully developed uteri filled with ova, or branched uterine structures. To identify the tapeworm species, we conducted molecular analysis using the proglottid segments. Genomic DNA was extracted using the QIAamp DNA Mini Kit (Qiagen, Hilden, Germany) and subsequently used as a template for polymerase chain reaction (PCR). The mitochondrial cytochrome *c* oxidase subunit I (*cox1*) gene and partial sequences of elongation factor-1 alpha (*ef1a*) were targeted for PCR amplification. The sequences of the PCR primers used were: T1F (5’-ATATTTACT TTAGATCATAAGCGG-3′) and T1R (5’-ACGAGAAAATATATTAGTCATAAA-3′) for *cox1*, and Tae_ef1/F4 (5’-TGTGGTGGAATCGATAAAAGG-3′) and Tae_ef1/R4 (5’-TCGATCTCATGTCACGAACG -3′) for *ef1a* [11, 12]. PCR was carried out using a 30 μL reaction mixture containing 15 μL Smart 2 × PCR Pre-Mix (SolGent Co., Ltd., Daejeon, Korea), 2 μL template DNA, 10 μM of each primer, and 11 μL distilled water, as described in a previous study [13]. The amplification process comprised 35 cycles of denaturation (94 °C for 30 s), annealing (60 °C for 30 s), and extension (72 °C for 80–90 s). The PCR products were sent to Macrogen (Korea) for direct sequencing using the same PCR primers. The 480-bp *cox1* sequence had a 100% match with the *T. saginata* sequence and a 94.8% match with the *T. asiatica* sequence. The 1078-bp *ef1a* sequence also had a 100% match with the sequence of *T. saginata,* a 99.3% match with the sequence of *T. asiatica* and a 95.3% match with the sequence of *T. solium*. The three *Taenia* species that infect humans had nucleotide differences at 73 and 57 polymorphic sites for the *cox1* and *ef1a* sequences, respectively (Additional file 1). Of these polymorphic sites, three sites (nucleotides 294, 336, 405) in *cox1* differentiated the three species. DNA sequences were aligned using the CLUSTAL W computer program [14]. Phylogenetic trees were constructed using the neighbor joining method [15] and genetic distances were computed using the Tamura-Nei method using the Geneious computer program (Fig. 1b and c). The neighbor joining tree used GenBank sequences derived from samples collected in Asia, and indicated that our specimen was in the same phylogenetic group as *T. saginata,* but not in the same group as *T. asiatica* or *T. solium.* In addition to molecular analysis, we also examined the patient’s stool specimen using the formalin-ether concentration method. However, we could not observe any *Taenia* species ova on the 10 slides examined. Ova were not observed inside the proglottids either, indicating that these proglottids were immature. Instead, *C. sinensis* ova were observed on one slide (Fig. 1d). Conversely, the level of serum IgG specific to *C. sinensis* measured using Enzyme-Linked Immunosorbent Assay (ELISA) was within the normal range. He was treated with praziquantel at the recommended dose of 25 mg/kg three times daily for 3 days [16]. After treatment, no proglottids or ova of *C. sinensis* were found in his stool.

## Discussion and conclusions

Human taeniasis is usually diagnosed by observing ova or gravid proglottids in the patient’s stool. From diagnoses conducted after 1993, the *Taenia* tapeworms infecting humans in Korea were identified as *T. solium, T. saginata*, and *T. asiatica* [5, 17, 18]. However, additional differential modalities may be required to clearly distinguish among these species, as morphological characteristics, such as the presence of an unarmed rostellum on the scolex of adult, the large number of uterine twigs, and the presence of a posterior protuberance, can be difficult to observe in individual strobili [17, 18]. Directe sequencing of *cox1* sequences have been used to identify the species causing human taeniasis. Cho et al. successfully used *cox1* to distinguish between *T. saginata* and *T. asiatica* [11]. Recently, studies conducted in China and Lao People’s Democratic Republic reported that hybridization between the three *Taenia* species cab iccyr, based on sequencing nuclear and mitochondrial genes [19, 20]. In our case, as we did not observe uterine structures or ova in the proglottids in our patient’s stool sample, these proglottids were likely immature. As such, we were unable to identify the tapeworm to the species-level using morphological characters, and thus sequenced both the nuclear gene, *ef1a,* and the mitochondrial gene, *cox1,* from the proglottid sample as well. Matches at several polymorphic sites confirmed that the *Taenia* specimen in this case is most likely *T. saginata,* and closely related to *T. saginata,* specimens sampled previously in Korea, China, Indonesia, Thailand and other countries (Fig. 1b and c). Here, we diagnosed that this case of human taeniasis was caused by *T. saginata.* We confirmed that tapeworm species by sequencing nuclear and mitochondrial gens, which successfully differentiated *T. saginata* from other *Taenia* species. Notably, we discovered that co-existence of taeniasis in a patient with subclinical clonorchiasis using a combination of diagnostic tools. Even in the era of molecular tools, the expertise in microscopy is still essential to achieve a correct diagnosis. Further, subclinical clonorchiasis in this case was confirmed only by examining the stool sample using microscopy. Regarding the use of ELISA to diagnose the presence of *C. sinensis,* ELISA results should be interpreated with caution, as the result can be negative even with an egg-positive [21, 22].

Early documentation of human taeniasis in Korea reported that the prevalence of *Taenia* was ranging from 7.2 to 12.0% [8–10]. More recently (2004–2008), the prevalence had declined to low levels of 0–0.01% [3, 23]. Since 2008, there has only been one study that documented four cases of *T. saginata,* which were diagnosed by sequencing of *cox1* [11]. Some researchers have suggested that human taeniasis is now close to absent in Korea, but cases of infection may be hidden, especially in areas where taeniasis was previously ubiguitous. In our case, the patient was from Jeollanam-do, the province with the second highest prevalence of taeniasis after Jeju-do (Island) according to the 1st Nationwide Survey [2]. Co-infection with other parasites can frequently occur in areas with high parasite prevalence, even though the helminthes do not share the same intermediate host [16]. Moreover, people who enjoy eating both raw beef and freshwater fish, as with our patient, have a higher chance of co-infection. The habit of eating raw fish or beef is deeply rooted in traditional customs among residents of rural areas of Korea [4]. In general, educating the public about not eating raw beef and freshwater fish is important for reducing the incidence of food-borne parasitic infections in Korea.

Initially, the patient took albendazole obtained from a pharmacy instead of visiting a hospital, although the medication was not effective. This may be due in part to the National Deworming Campaign in Korea over the past years that focused on soil-transmitted helminthiases and highlighted repeated administration of albendazole to control parasitic infections. An initial 600-mg tablet of praziquantel (10 mg/kg) may be sufficient for treating taeniasis in this case. The patient also received additional praziquantel at a dosage of 75 mg/kg/day for 3 days to treat clonorchiasis. Although we could not find scolex of *Taenia* species in the submitted specimens, the treatment may have been effective in controlling both taeniasis and clonorchiasis.

In this study, we described an unusual case of co-infection of *T. saginata* in a patient with subclinical clonorchiasis in Korea. Our results indicate that diagnosis through molecular methods may be helpful in cases with ambiguous morphological characters such as immature proglottids. Overall, a combination of diagnostic tools should be used for the accurate diagnosis of subclinical parasitic infections.

## Additional file

Additional file 1: Polymorphic sites of *cox1* and *ef1a* sequences of the submitted proglottids compared with those of human *Taenia* tapeworms. Numbers indicate the positions from 5′end of the *cox1* or *ef1a* gene, and dots indicate sequence matches with *T. saginata.* *Three sites (nucleotide 294, 336, 405) were distinguishable substitiutions in all of three human *Taenia* tapeworms. (XLSX 16 kb)

## Abbreviations

*cox1*: Cytochrome *c* oxidase subunit I
*ef1a*: Elongation factor-1 alpha
ELISA: Enzyme-linked immunosorbent assay
PCR: Polymerase chain reaction

## References

[1] Hotez PJ, Brindley PJ, Bethony JM, King CH, Pearce EJ, Jacobson J (2008). Helminth infections: the great neglected tropical diseases. J Clin Invest.

[2] Ministry of Health and Social Affairs and Korean Association for Parasite Eradication. Prevalence of intestinal parasitic infections in Korea. The 1th report. Seoul, Korea. 1971. 1–26. (in Korean).

[3] Korea Centers for Disease Control and Prevention. National survey of intestinal parasitic infections in Korea, 8th report 2013. KCDC 2014;1:89–94. (in Korean).

[4] Cho SH, Lee KY, Lee BC, Cho PY, Cheun HI, Hong ST, Sohn WM, Kim TS (2008). Prevalence of Clonorchiasis in southern endemic areas of Korea in 2006. Korean J Parasitol..

[5] Cho PY, Na BK, Choi KM, Kim JS, Cho SH, Lee WJ, Lim SB, Cha SH, Park YK, Pak JH, Lee HW, Hong SJ, Kim TS (2013). Development of a polymerase chain reaction applicable to rapid and sensitive detection of *Clonorchis sinensis* eggs in human stool samples. Pathog Glob Health.

[6] Chai JY (2013). Human taeniasis in the Republic of Korea: hidden or gone?. Korean J Parasitol..

[7] Dorny P, Praet N (2007). *Taenia saginata* in Europe. Vet Parasitol.

[8] Kobayashi H, Kwon N (1917). Studies on the intestinal parasites of Koreans: first report. J Chosen Med Ass..

[9] Kojima R, Ko T (1919). Researches on intestinal parasites of Korea in south Gyeongsang-do, especially on the distribution of the liver fluke. J Chosen Med Ass..

[10] Uchida R (1924). Results of fecal examination on intestinal helminths among Korean prisoners in the Seodaemoon jail. J Chosen Med Ass.

[11] Cho J, Jung BK, Lim H, Kim MJ, Yooyen T, Lee D, Eom KS, Shin EH, Chai JY (2014). Four cases of *Taenia saginata* infection with an analysis of *COX1* gene. Korean J Parasitol.

[12] Okamoto M, Nakao M, Blair D, Anantaphruti MT, Waikagul J, Ito A (2010). Evidence of hybridization between *Taenia saginata* and *Taenia asiatica*. Parasitol Int.

[13] Jeon HK, Kim KH, Eom KS (2011). Molecular identification of *Taenia* specimens after long-term preservation in formalin. Parasitol Int.

[14] Thompson JD, Higgins DG, Gibson TJ (1994). CLUSTAL W: improving the sensitivity of progressive multiple sequence alignment through sequence weighting, position-specific gap penalties and weight matrix choice. Nucleic Acids Res.

[15] Saitou N, Nei M (1987). The neighbor-joining method: a new method for reconstructing phylogenetic trees. Mol Biol Evol.

[16] Hong ST, Rim HJ, Min DY, Li X, Xu J, Feng Z, Lee SH (2001). Control of clonorchiasis by repeated treatments with praziquantel. Korean J Parasitol.

[17] Eom KS, Rim HJ (1993). Morphologic descriptions of *Taenia asiatica* sp. n. Korean J Parasitol..

[18] Eom KS, Rim HJ (2001). Epidemiological understanding of *Taenia* tapeworm infections with special reference to *Taenia asiatica* in Korea. Korean J Parasitol..

[19] Yamane K, Suzuki Y, Tachi E, Li T, Chen X, Nakao M, Nkouawa A, Yanagida T, Sako Y, Ito A, Sato H, Okamoto M (2012). Recent hybridization between *Taenia asiatica* and *Taenia saginata*. Parasitol Int.

[20] Sato MO, Sato M, Yanagida T, Waikagul J, Pongvongsa T, Sako Y, Sanguankiat S, Yoonuan T, Kounnavang S, Kawai S, Ito A, Okamoto M, Moji K (2018). Taenia solium, Taenia saginata, Taenia asiatica, their hybrids and other helminthic infections occurring in a neglected tropical diseases' highly endemic area in Lao PDR. PLoS Negl Trop Dis.

[21] Kim YJ, Lee SM, Choi GE, Hwang SH, Kim HH, Lee EY, Chang CL (2010). Performance of an enzyme-linked immunosorbent assay for detection of *Clonorchis sinensis* infestation in high- and low-risk groups. J Clin Microbiol.

[22] Han S, Zhang X, Wen J, Li Y, Shu J, Ling H, Zhang F (2012). A combination of the Kato-Katz methods and ELISA to improve the diagnosis of clonorchiasis in an endemic area. China PLoS One.

[23] Chai JY, Park JH, Guk SM, Kim HJ, Kim WH, Kim JL, Gu YS, Shin EH, Park HM, Hong KS, Kim SD, Lee SH (2006). Status of intestinal parasite infections among 4,137 residents from provinces nationwide and metropolitan areas in the Republic of Korea (2004). Infect Chemother.

